# The Effects of Camphor on Vegetables

**Published:** 1840-09

**Authors:** 


### EFFECTS OF CAMPHOR ON VEGETABLES.

"The stimulant effects of camphor upon the human and some other animal bodies are well known; but those on vegetables are not only new, but astonishing in their nature. A piece of the woody stem of the tulip-tree, with one flower and two leaves, taken out of a pot of water, containing several other flowers of the same plant, all, to appearance, in the same state, was placed in eight ounces of water, which had been stirred up for some time with one scruple of good camphor. In a little while, an unusually lively appearance became remarkable in the flower in the camphor; while the others, though they had the benefit of a larger quantity of water, were sensibly drooping.

"The two leaves first elevated themselves considerably on their foot stalks; the flower expanded more than in a natural state; the stamina occhives receded from the pistillum; and the three leaves of the calix, or flower-cup, were remarkably reflected back, and grew extremely rigid and elastic. The internal surface of the petals of the flower perspired considerably, though a similar perspiration could not be perceived in the flowers of the same room and temperature. The camphorated plant continued in a very invigorated state for two whole days, after which it began to droop; but the leaves drooped and decayed sooner than the flower. The other flowers and leaves of the tulip-tree left in simple water, did not live more than half as long as that in the water impregnated with camphor.

"Notwithstanding these surprising effects, no odor of camphor could be traced inany part of the branch, except what was immersed in the fluid. This circumstance seems to render it probable that the camphor was not absorbed by the plant, but that it exerted its remarkable influence entirely through the solids to which it was immediately applied. The appearance, however, was very striking, and might be compared to the beneficial effects of opium on the human constitution. Several other experiments were made with camphor on plants, in all of which was very evident that camphor operated as a powerful and wholesome stimulant. A stalk of yellow iris, with one expanded flower, was taken out of a phial of water in which it had been placed more than a day.

"The flower had begun to droop; but in a very few minutes after being put in a phial of the same size, containing a few grains of camphor, it began to revive, and continued in a vigorous state for many hours. As camphor is but very sparingly soluble in water, it is natural to conclude that the stimulant effects were produced by a very small part of the quantity mingled with the water. This discovery might induce us to make experiments with camphor as a manure, if the expense of trying them on a scale sufficiently large were not excessive. But still, we may apply the camphor in the manner before mentioned; and can that be termed a useless purpose? A few grans of camphor acting as a cordial, will revive a drooping plant, increase its beauty and prolong its existence. In the eye of the florist, these are objects of no mean importance."—*Burt's Observations on the Curiosities of nature.*

REMARKS.

The foregoing extract is from a late number of the National Gazette, published in Philadelphia. The editor of that excellent paper will confer a favor on the writer of these brief and hasty *remarks*, by an early republication of them in his valuable columns.

However interesting or "astonishing in its nature" may appear the fact, that "camphor produces stimulant effects on vegetables," the knowledge of that fact is far from being "*new*." It is certainly so old as to be a product of the last century; whether any older, we are not prepared to say.

The question was once made a subject of debate or conversation in the Philadelphia Medical Society, we think during the sessions of that Institution, in the winter of 1796, '97 or '98; and in the course of the next spring and summer, Dr. Caldwell, then a resident member of the Society, closed the controversy, if such it was, by a series of experiments satisfactory to himself, as well as to others.

The vegetables on which the Doctor chiefly experimented, were the common Althea, and a species of the Magnolia; and their flower-buds were the parts he especially selected as his subjects.

In the course of his experiments he sometimes lopt from the plant the twigs containing the buds, and immersed them in water holding camphor in solution; and at other times, suffering the twigs to remain on their parent-limbs, he wrapt around them a few folds of cloth, or small bundles of cotton, which he kept constantly moist with the camphor-solution. And, in every instance, the growth and development of the bud was hastened and invigorated, in a remarkable degree. The following was his mode of proceeding.

He selected on the same plant, several twigs containing flower-buds alike in age, healthfulness, and vigor. The twigs moreover were on parts of the plant remote from each other, in order that those of them whose buds were to serve as standards of natural development, might not be acted on by the camphor-exhalation.

Some of these twigs he moistened with the camphor-solution, some with water holding in solution carbonic acid, others with river or rain-water, and the remainder he left to the action alone of the atmosphere, and its native humidity, whether of rain or dew. And the results of his experiments were exceedingly uniform.

The camphorated buds always expanded with far the highest degree of activity. Next in activity were those wet with acidulated water. Still less rapid was the development of the buds moistened with rain or river-water. And when the atmosphere contained but little moisture, the growth and expansion of those not moistened at all were the least active and vigorous of the whole. Nor was this stimulating action of camphor and carbonic acid confined to the flower-buds. The *leaves* of the moistened twigs were similarly influenced by it; though not perhaps to the same extent.

In another course of experiments, by the same gentleman, assafœtida was employed, as the stimulant material, with similar results. The flowers and leaves were uniformly quickened by its action, in their expansion and growth. Nor was this all. The gentle stimulation of electricity produced also on vegetables invigorating effects. They grew under its influence, and put forth their flowers, with superabundant activity.

To the confirmation and establishment of the same principle, the following experiment farther contributed. Three small grass-patches, distant from each other, were selected. One of them was watered with a camphor-solution, another with a weak solution of assaœtida, and the third with common water; and the results were analogous to those already stated. The grass of the two spots watered with the stimulating solutions grew and flourished with *unusual*, the other with only *common* luxuriance and rapidity.

Shortly after the foregoing facts became known, the late Professor Barton instituted a series of similar experiments, with corresponding results. And, a year or two afterwards, Dr. Church, late of Philadelphia, wrote and published an Inaugural Dissertation, on the same subject. The experiments detailed by him were numerous and well conducted; and their issues were identical with those of the preceding experiments, The growth of vegetables was always hastened by the action of the several stimulants he employed.

The dissertation by Dr. Church is no doubt to be found in the Library of the Pennsylvania Hospital; and it is believed to contain a note from Dr. Caldwell, on the effects of stimulation on vegetable growth.

At this period Dr. Caldwell was engaged in the study of *Philosophical* Botany, especially of the physiology and habits of plants. On certain *night-sleeping* plants he made some experiments not perhaps altogether unworthy of notice. His object was to prevent them from sleeping, which he attempted to do in the following manner.

The somnolent action or rather *inaction* of those plants is known to commence with the commencement of twilight, or a little earlier. To bar their slumbers therefore, the doctor, just before their leaves began to "droop and drowse," commenced on them his process of camphor or assafœtida-stimulation; and, as twilight descended and was just beginning to thicken about them, he added the stimulant action of *artificial light.* This he did, by placing around the plants experimented on, a number of powerfully reflecting lamps, lit up to the highest pitch of brilliancy. And though the result did not quite equal his expectation, it did so to a considerable extent. The stimulation employed rendered the sleep of some of the more sensitive plants, especially we think of the young locust, the mimosa sensitiva, and the eastern acacia, considerably less profound than usual.

But how, we ask, is it possible for these things to be otherwise?

The analogies between vegetables and animals are much greater and more numerous, and the differences between them immeasurably less, than than they are generally supposed to be. So true is this, that no naturalist has yet been able to say, with positive definiteness, where the one class of beings ends, and the other begins.

Vegetables, in common with animals, are organized, living, and susceptible beings—susceptible we mean of vital impression, and capable of vital action and growth. By no physiologist will these positions be contradicted or questioned. They are axioms in the science of organization anp life. Every vital action moreover, whether in vegetables or animals, is as certainly and obviously the product of stimulation, as the descent of a falling body is the product of gravity. This we repeat is an axiom in physiology.

It is in consequence of the stimulation of heat and light and food and moisture, that vegetables take on action and growth in the spring. For food stimulates them, and produces vital action in them, as certainly as it does in relation to animals. And a leading reason why vegetables grow more vigorously, rapidly, and with greater luxuriance in tropical, than they do in temperate and frigid climates, is because they are more abundantly stimulated by heat.

In a word; it is pride and ignorance, much rather than a knowledge of nature, and a correct and becoming sense of his own standing, that inflates man with the vain but flattering belief, that he is demigod or a god, compared with a mushroom. A more thorough knowledge of things as they are, than he now possesses, will yet convince him that the real difference is much less than he now imagines it.

This view of things is greatly strengthened by the late observations of M. Turpin, communicated to the Academy of Science, in Paris, in the course of which he discovered the actual *transformation of globules of milk* into a peculiar species of *mucor or mould.* The mucor he contends did not spring out of the globules, as plants do out of the ground in which they are rooted. They were transformed and converted into the mucor, as seeds are into the plants which they produce. The globules moreover, having given birth to the mould, retained their formal existence no longer, but entirely disappeared, as seeds do after the birth and temporary nourishment of their plants. C. C.

